# Attitude of literate married men towards the use of aphrodisiac herbs in Ilorin, Kwara state, Nigeria

**DOI:** 10.4314/ahs.v22i2.49

**Published:** 2022-06

**Authors:** Aminat Adeola Odebode, Issa Baba Awoye

**Affiliations:** 1 Department of Counsellor Education, Faculty of Education, University of Ilorin, Nigeria; 2 Department of Behavioural Sciences, College of Health Sciences, University of Ilorin, Nigeria. https://orcid.org/0000-0002-7491-7167. Tel: +2348033559293. Issababa2002@yahoo.com

**Keywords:** Attitude, Literate Married Men, Aphrodisiac Herbs, Nigeria

## Abstract

**Background:**

Sexual dysfunction among men is on the increase in Nigerian societies; however, many do not seek health care; and may prefer using aphrodisiac herbs.

**Objectives:**

We investigated the attitude of literate married men towards the use of aphrodisiac herbs in Ilorin, Kwara State, Nigeria. We also examined whether there would be a significant difference in the attitude of literate married men towards the use of aphrodisiac herbs based on age and educational attainment.

**Methods:**

Descriptive survey method was adopted for the study. A total of 200 literate married men were selected through simple random and purposive sampling. The participants responded to a researcher-designed questionnaire. The data were analysed using descriptive and inferential statistics at 0.05 alpha level.

**Results:**

The attitude of literate married men was positive. There were significant differences in the attitude of literate married men towards the use of aphrodisiac herbs in Ilorin, Kwara State based on age and educational attainment.

**Conclusions:**

Most literate married men in Ilorin, Kwara State, Nigeria had a positive attitude towards the use of aphrodisiac herbs. Doctors and counsellors should organise community-based talk to orientate literate married men on the use of aphrodisiac herbs.

## Introduction

Sexual function is a fundamental part of human identity and how humans feel. Sexual function is linked to sexual satisfaction^1^. Sexual satisfaction is a feeling that an individual obtains through mental and physical enjoyment of sexual intercourse. Sexual health ranges from physical, psychological to socio-cultural well-being about sexuality^1^. A person's sexual health is a critical factor in determining the capacity for maintaining healthy relationships^2^, ^3^.

Sexual dissatisfaction could lead to sexual deprivation and vice versa among spouses which in turn can lead to extramarital affairs.^4^ Sexual dysfunction could be a disguised manifestation of other underlying conditions, such as hypertension, diabetics, heart failure and others^5^. Couples suffering from sexual dysfunction are more likely to suffer from anxiety, depression and low self-esteem^6^. This implies that a healthy intimate relation rests on the sexual relationship of the two partners, while low sexual function can split the intimate relation.

It was reported in some studies that, about 15 to 41% of men suffer from sexual dysfunction or are not satisfied with sex life^7^, ^8^. It has, also, been reported that between 40–60% of men in Pakistan, Egypt, and Nigeria experience varying degrees of sexual dysfunction^9^,^10^. While, sexual dysfunction is common among men, accurate data on sex-related matters seem not to exist globally and this has led to under treatment^11^. It is not unlikely that men in Nigeria may not discuss sexual dysfunction because of their belief about discussing sex. This belief may be a function of cultural orientation and background which emphasizes the hidden nature of heterosexual relationships. It is worthy of note that the significant disorder of sexual dysfunction in men is premature ejaculation, erectile dysfunction and Hypoactive Sexual Desire Disorder (HSDD)^12^, ^13^. Of these three types of sexual dysfunction, premature ejaculation is likely the most prevalent sexual dysfunction according to the results of numerous epidemiological study^13^, ^14^, ^15^. Overall, the prevalence rate of premature ejaculation falls somewhere between 25 and 40% in the global population of men across all age groups^15^. Estimates vary, but, overall, 1 out of 3 men may be affected by premature ejaculation at some time in their life^16^, ^17^.

In the course of improving sexual performance, some married men have chosen to use aphrodisiac herbs as a source of intervention. The use could be due to the men's unwillingness to discuss sexual issues with doctors and their dislike for drug-mediated erections. Invariably, men believe in the efficacy of aphrodisiac herbs^18^, and perhaps to keep their sexual dysfunction from being heard by the third party. Aphrodisiac herbs are libido boosters or sexual performance enhancing herbs which are also known as “manpower, body energizer or action pill”^16^. The Hausa and Yoruba people in Nigeria call it ‘qurantanshi’ and ‘Ogun Aleko’ (potent booster for sexual performance). The aphrodisiac is getting increasingly popular amongst young adults and sexually active men to enhance their sexual ability^5^. Aphrodisiac herbs are increasing in society because every woman expects that their men are “capable”sexually^19^. Aphrodisiac herbs are prepared in different forms. There are local variants such as a mixture of local gin and herbs (Agbo Gbogbonise, Sepe or Paraga). There are also well packaged industrially made variants in packets of pills, or tablets such as “Spanish fly, Enpulse, Vimax, Virillis, M-Energex, High T, Male X” and those in liquid forms such as Alomo bitters among others.^20^ The use of herbal medicines has substantially increased due to escalated prevalence and impact of sexual problems worldwide and estimates predicting the incidence to raise over 320 million^21^ by the year 2025.

Attitudes emanate from a Latin word ‘aptitudo’ which means fitness-fitness to perform a particular task. The term is used to connote acting in a way towards some aspects of one's environment. It is a positive or negative assessment of objects, ideas, events, people or anything^22^. This implies that attitude is a human tendency to judge an entity with favor or disfavor. In essence, an attitude refers to human's effect towards a particular phenomenon. Sex is a way by which men express their status and feelings; many men want to impress their partner and aphrodisiac being a sex enhancer, could attract positive attitude from men. On the other hand, a non-medical drug, may be perceived with a negative attitude. It is against this background that this study investigated the attitude of literate married men towards aphrodisiac herbs use in Ilorin, Kwara State, Nigeria.

## Problem

The desire for intimacy and sexual gratification is a global phenomenon and has remained life-long expressions among couples. However, the prevalence of sexual dysfunction among men continues to increase, and they neither enjoy their sex life nor satisfy their partners. Sadly, many men do not present sexual dysfunction for medical treatment; they prefer to use sexual boosters such as aphrodisiac herbs and other self-medications. Worse still, sexual dysfunction has caused unhappiness among couples, low self-confidence in men and generally tensed atmosphere for children. Various studies have been conducted on aphrodisiac herbs which are partly related to the focus of this study^5^, ^21^, ^23^, ^24^, ^25^, ^26^, ^27^. However, most of this research centered on reproductive perspectives, women respondents, erectile dysfunction or premature ejaculation^4^,^6^,^7^,^8^ and considered different locations. Thus, the present study was conducted to bridge the research gap.

The study was designed to answer the following research question: What is the attitude of literate married men towards the use of aphrodisiac herbs in Ilorin, Kwara State, Nigeria?

## Methods

### Design

Descriptive survey and analytical methods were used for the study.

### Participants and Setting

Nigeria is a multilingual country having 36 States; the country is also divided into six geo-political zones that comprise the 36 States. Kwara State is one of these States and belongs to the North Central geo-political zone while, Ilorin is the capital of Kwara State.

The population of this study consists of all literate married men in Kwara State, with a projected population^28^ of 1,193,783. Using Epi Info Version 7 with an expected frequency of 50% of literate married men with a positive attitude, at 95% confidence intervals and 6.0% margin of error. A design effect of 1 was used because random sampling method was used. A sample size of 188 was obtained. For non-responses, 10% was added, and the final sample was 217. A 2-stage sampling technique which comprises random and purposive sampling techniques were employed to select the sample. At stage one, simple random sampling technique was used to select one Local Government Area (LGA) out of the three Local Government Areas that constitute Ilorin metropolis. At stage 2, purposive sampling technique was adopted to select literate married men from Five Ministries, Eight Schools and one LGA secretariat. This procedure continued until 206 literate married men were sampled for the study. The age range of the respondents was in 3 age brackets: 20–30 years; 31–40 years; & 41 years and above). Similarly, the educational attainment of the respondents cuts across Primary School Certificate/Secondary School Certificate (PRY/O'level); Nigeria Certificate in Education/Ordinary National Diploma (NCE/OND); Higher National Diploma/First Degree (HND/1st Degree); and Postgraduate Degree. The study was conducted between February and June 2019.

### Instrumentation and Data Collection

The instrument that was used for data collection in this study was a researcher-designed questionnaire entitled “Attitude towards Aphrodisiac Herbs Use Questionnaire (AAHUQ)”. The questionnaire consisted of two sections. Section “A” elicits information on the demographic data of the respondents, i.e. age and educational attainment. Section “B” was designed to collect information on “attitude towards aphrodisiac use”, and it consists of 20 items. Section B was scored using 4 points Likert rating scale of strongly agree (4 points) to strongly disagree (1 point); the benchmark weighted mean of 2.50 was derived from this (4+3+2+1/4). Thus, a weighted mean score of 2.50 and above was regarded as a positive attitude towards the use of aphrodisiac herbs. In contrast, a weighted mean score of less than 2.50 was regarded as a negative attitude towards the use of aphrodisiac herbs. Experts in Measurement and Evaluation validated the instrument. The researcher employed tests re-test reliability method, and a coefficient of 0.60 was obtained; the instrument was therefore considered reliable enough for this study. Similarly, the instrument was pre-tested on 20 literate married men in other LGAs that were not selected for the study to ensure the removal of ambiguous items for the respondents. The researcher administered the questionnaire forms with the help of two research assistants who have been briefed on the purpose of the study. The questionnaires were collected immediately after completion to ensure high return. A total of 206 questionnaire forms were administered, but only 200 copies were retrieved back. This made a 97% return rate of the instrument.

### Ethical Issue

Ethical approval (REF: UITH/CAT/189/19A/008) was sought and granted by the Ethical Review Committee (ERC), University of Ilorin Teaching Hospital, Nigeria. Furthermore, the researcher sought the consent of the selected participants by explaining in details the purpose of the study. The researcher attached informed consent forms to the questionnaire, and with the two research assistants, administered the paper instrument. The participants were assured that their responses would be used for research purpose only, that there was minimal risk/harm or force in participating in the study, and they could opt-out at any time they wish. The participants were not asked to write their names or addresses on the questionnaire form to ensure anonymity.

### Data Analysis

The data were manually checked for any error, entered to epidata and edited before being moved to SPSS V. 22.0 for analysis. Percentage, means, standard deviation and Analysis of Variance (ANOVA to compare the means of three, or more groups) statistics were employed to analyze the data. A p-value of less than 0.05 was taken as significant.

## Results

### Demographic Information

The demographic data were analyzed using descriptive statistics of percentage. Out of the 200 respondents that returned the questionnaire forms, 35 (17.5%) were between 20–30 years, 33 (16.5%) were between 31–40 years old while, 132 (66.0%) were between 41 years and above. A total of 33 (16.5%) of the respondents have Primary/O' level certificate, 32 (16.0%) have NCE/OND degree, 131 (65.5%) have HND/1st degree, while, 4 (2.0%) have Postgraduate degree.

### Research Question

What is the attitude of literate married men towards use of aphrodisiac herbs in Ilorin, Kwara State, Nigeria?

Attitude of literate married men towards use of aphrodisiac herbs is shown in Table 1. The weighted mean score of 2.68 is greater than the bench mark weighted mean of 2.5 thus, the attitude of literate married men in Ilorin, Kwara State, Nigeria towards use of aphrodisiac herbs is positive.

**Table 1:** Mean and Rank Order of Attitude of Literate Married Men towards Use of Aphrodisiac Herbs

Item No.	As a literate married man, I feel that:	Mean	Rank
13	physicians should be enjoined to prescribe aphrodisiac herbs for their patients diagnosed to be in need of such	3.35	1^st^
3	aphrodisiac herbs should be developed to become branded products	3.23	2^nd^
15	sponsored advert on aphrodisiac herbs use as a therapy for correcting sexual dysfunction should be upheld among other expressions.	3.22	3^rd^
18	I can make aphrodisiac herbs if I am taught how to prepare it	3.05	4^th^
16	using aphrodisiac herbs vigorously enhances a man's sexual capacity	3.03	5^th^
7	I regularly need to use aphrodisiac to stimulate my sexual urge	3.01	6^th^
5	aphrodisiac have many sexual health benefit	2.89	7^th^
20	men of different religious background can use	2.88	8^th^
8	one does not need to be shy of patronizing aphrodisiac herb sellers	2.87	9^th^
1	aphrodisiac herbs are better than the orthodox sexual performance medicine	2.85	10^th^
9	I used to encourage my friends to use aphrodisiac herbs to enhance their sexual prowess	2.82	11^th^
10	recommending aphrodisiac herbs for my wife is ok	2.71	12^th^
11	sexual dysfunction can be dealt with appropriately using aphrodisiac herbs	2.70	13^th^
12	every married man should use aphrodisiac herbs to correct their sexual dysfunctions	2.68	14^th^
19	occupational type should not be a barrier to the use of aphrodisiac herbs	2.66	15^th^
2	sexual herbal products pose no wide spread risk	2.64	16^th^
6	hospitals or pharmacies should stock sexual enhancement herbal medicine	2.61	17^th^
14	aphrodisiac herb sellers should be allowed in different motor parks or joints	1.50	18^th^
4	NAFDAC do not need to regulate herbal product for people's use	1.46	19^th^
17	consuming aphrodisiac herbs at least three times in a week before having sexual intercourse is not bad	1.40	20^th^
**Weighted Mean Score**		**2.68**	

Table 2 indicates that the calculated F-ratio is 135.25 while the critical F-ratio is 3.00. Since the calculated F-ratio of 135.25 is greater than the critical F-ratio of 3.00 at p-value less than 0.05.

**Table 2:** Analysis of Variance (ANOVA) on the Attitude of Literate Married Men towards Use of Aphrodisiac Herbs Based on Age

Age Diff.	SS	df	MS	Cal. F-ratio	Crit. F-ratio	P-value
Between group	17906.190	2	8953.095	*135.25	3.00	0.01
Within group	13040.565	197	66.196			
Total	30946.755	199				

* P<0.05

Table 3 shows that all the groups differed from one another, but the significant difference noted was as a result of the mean of Group 3 that has the highest mean of 60.89, hence the significant difference noted in the ANOVA on Table 2 was brought about by literate married men who are between 41years & above.

**Table 3:** Duncan Multiple Range Test (DMRT) Showing the Difference in the Attitude of Literate Married Men towards Use of Aphrodisiac Herbs Based on Age

Age	N	Mean	Group	Duncan Groupings
20–30 years	35	35.77	1	A
31–40 years	33	51.61	2	B
41years& Above	132	*60.89	3	C

Table 4 indicates that the calculated F-ratio is 3.76 while the critical F-ratio is 2.60. Since the calculated F-ratio of 3.76 is greater than the critical F-ratio of 3.00 at p-value less than 0.05.

**Table 4:** Analysis of Variance (ANOVA) on the Attitude of Literate Married Men towards Use of Aphrodisiac Herbs based on Educational Attainment

Educational Attainment	SS	df	MS	Cal. F-ratio	Crit. F-ratio	P-value
Between group	1685.346	3	561.782	3.76*	2.60	0.00
Within group	29261.409	196	149.293			
Total	30946.755	199				

* P<0.05

Table 5 shows that significant difference noted was as a result of the mean of Group 1 that has the highest mean of 67.91, hence the significant difference noted in the ANOVA on Table 3 was brought about by literate married men who have Pry/O’ Level Certificate.

**Table 5:** Duncan Multiple Range Test (DMRT) Showing the Difference in the Attitude of Literate Married Men towards Use of Aphrodisiac Herbs Based on Educational Attainment

Educational Attainment	N	Mean	Group	Duncan Groupings
Pry/O' Level Certificate	33	*67.91	1	A
NCE/OND	32	55.57	2	B
HND/1^st^ Degree	131	51.61	3	C
Postgraduate Degree	4	39.25	4	D

## Discussion

The findings of this study showed that the attitude of literate married men in Ilorin, Kwara State, Nigeria, towards the use of aphrodisiac herbs was positive. This finding is consistent with a previous finding conducted in South-Eastern region of Nigeria, which revealed that many men have a positive attitude towards the use of aphrodisiac herbs as an alternative to medically prescribed drugs; besides, men have a strong belief in the efficacy of aphrodisiac herbs5. Similarly, this finding agrees with earlier findings which showed that men have a positive attitude towards the use of aphrodisiac to enhance their sexual ability^12^, ^29^, ^30^. Conversely, the finding of a study conducted in Mumbai, India is not in line with an earlier finding that men do not attach much importance to a sexual relationship; thus, they could have a negative attitude towards sex boosting herbs^31^. The reason for this finding could be that men derive pleasure in being sexually active; this makes them have an elevated level of confidence as they can exert themselves as men. Therefore, it is not unlikely that men would have a positive attitude towards any sexual boosting herbs.

There was a significant difference in the attitude of literate married men in Ilorin, Kwara State, Nigeria towards the use of aphrodisiac herbs based on age. This finding corroborates a previous result of a study carried out in an infertility clinic in Uganda which revealed that age is significant in the attitude towards the use of aphrodisiac among men^21^. Similarly, the findings agree with an earlier finding in South-Eastern region of Nigeria 5 that significant association exists between age and men's attitude towards the use of aphrodisiac herbs. Although, younger men also expressed a positive attitude towards the use of aphrodisiac, those aged 41 years and above expressed a more positive attitude towards the use of aphrodisiac herbs. This finding could be due men not seeing age as a barrier to sexual relationships.

There was a significant difference in the attitude of literate married men in Ilorin, Kwara State, Nigeria towards the use of aphrodisiac herbs based on the level of educational attainment. This finding is in line with a previous study carried out among Unani & Ayurvedic health care seekers in Dhaka city which showed that educational attainment has a significant influence on the knowledge and attitude towards aphrodisiac among men^29^. On the other hand, this finding is not in support of a finding carried out in Kwara State, Nigeria that revealed no significant difference in the attitude towards the use of aphrodisiac herbs based on educational attainment^23^. Although, men who have higher educational attainment expressed a positive attitude towards the use of aphrodisiac, literate married men who have Primary/O' level Certificate expressed a more positive attitude towards the use of aphrodisiac herbs. This finding could be due to the exposure associated with higher educational degrees; thus, participants who are more educated may not be too disposed to the use of aphrodisiac herbs^29^. In another perspective, this finding could be because men derive pleasure in having sex and, the more agile they are sexually, the more satisfied they are; this means that they can go to any length irrespective of their educational attainment, to enhance their sexual performance such as taking aphrodisiac herbs^18^.

## Limitations of the Study

The study had some limitations that warrant caution when generalizing the findings. Firstly, a limited number of participants partook in the study; hence, their expressions may not apply to all men. Secondly, the moderating variables of this study were limited to age and educational attainment among many other demographics. Thirdly, that the participants expressed a positive attitude towards the use of aphrodisiac may not imply that they use the herbs. However, these limitations did not decrease the validity of this study.

## Conclusion

The attitude of literate married men Ilorin, Kwara State, Nigeria, towards the use of aphrodisiac herbs is positive. Furthermore, age and educational attainment significantly influenced the attitude of literate married men Ilorin, Kwara State, Nigeria, towards the use of aphrodisiac herbs.

## Recommendations

Positive attitude towards the use of aphrodisiac herbs may imply its use; therefore, married men should use aphrodisiac herbs that are certified by drug agencies and those that have a scientific basis for enhancing sexual satisfaction to avoid damage to body organs.

There is the need to include marriage and family in the health delivery team of the nation to counsel married men on the use of aphrodisiac herbs. Based on the positive attitude of literate married men in Ilorin, Kwara State, towards the use of aphrodisiac herbs, government, pharmacists and EFCC in Nigeria should call for quality assurance on aphrodisiac herbs to ensure safety and quality of the herbs to men for the treatment of sexual dysfunction.

Counsellors should be at the forefront to campaign for the inclusion of aphrodisiac herbs into the medical/drug prescription list for the treatment of sexual dysfunction among men to ensure the safe use of the herbs. In the future, it is neces0sary to look into the use of aphrodisiac herbs using a larger population of men and other moderating variables such as religion and the number of children.

## Role of authors

Odebode conceived the research idea, wrote the protocol. Issa monitored the data collection process. Odebode wrote the draft and led the entire writing process; Issa performed the quantitative analysis. Both authors gave thorough review all sections of the manuscript preparation.

## References

[1] Moreau C, Kagesten AE, Blum RW (2016). Sexual dysfunction among youth; an overlooked sexual health concern. BMC Public Health.

[2] Byers ES (2005). Relationship satisfaction and sexual satisfaction: a longitudinal study of individuals in long-term relationships. Journal of Sex Research.

[3] Yeh HC, Lorenzo FO, Wickrama KA (2006). Relationships among sexual satisfaction, marital quality, and marital instability at midlife. Journal of Family Psychology.

[4] Yanez D, Castelo-Branco C, Hidalgo LA, Chedraui PA (2006). Sexual dysfunction and related risk factors in a cohort of middle-aged Ecuadorian women. Journal of Obstetrics and Gynecology.

[5] Iwuozor OK (2019). Heavy metal concentration of aphrodisiac herbs locally sold in the South-Eastern region of Nigeria. Pharmaceutical Science and Technology.

[6] Akinloye OO, Yinusa R (2011). assessment of complementary and alternative medicine (CAM) usage to enhance male sexual performance in Ogbomoso metropolis. J Public Health Epidemiol.

[7] McGraw SA, Rose RC, Althof SE, Dunn M, Cameroon A, Wong D (2015). Perceptions of erectile dysfunction and phosphodiesterase type 5 inhibitor therapy in a qualitative study of men and women in affected relationships. Journal of Sex and Marital Therapy.

[8] Lo WH, Fu SN, Carlo S, Wong KH, Chen ES (2014). Prevalence, correlates, attitude and treatment seeking of erectile dysfunction among type 2 diabetic Chinese men attending primary care outpatient clinics. Asian Journal of Andrology.

[9] Shaeer KZM, Osegbe DN, Siddiqui SH, Razzaque A, Glasse DB, Jaguste V (2003). Prevalence of erectile dysfunction and its correlates among men attending primary care clinics in three countries: Pakistan, Egypt, and Nigeria. Int J Impot Res.

[10] Bolaji OO, Abiodun CJ, Sunday AA (2016). Prevalence of erectile dysfunction and possible risk factors among men of South-Western Nigeria: a population based study. Pan African Medical Journal.

[11] Bratus D, Bratus T (2020). Men with serious chronic illnesses and malignances are less likely to seek treatment for erectile dysfunction. International Journal of Impotence Research.

[12] Rosing D, Klebingat K, Berberich H, Hartmut AG, Loewit K, Bier KM (2010). Male sexual dysfunction: diagnosis and treatment from a sexological and interdisciplinary perspective. Dtsch Arztebl Int.

[12] Harti SS (2014). Survey on knowledge of reproductive and sexual health in the community and clinical evaluation of veeryasthambha Vati in premature ejaculation.

[13] Rosen R, Kountz D, Post-Zwicker T (2006). Sexual communication skills in residency training: the Robert Wood Johnson model. Journal of Sexual Medicine.

[15] Montague DK, Jarow J, Broderick GA (2004). AUA guideline on the pharmacologic management of premature ejaculation. Journal of Urology.

[14] Bello UL, Jibrin I (2015). Use of herbal medicines and aphrodisiac substances among women in Kano State, Nigeria. IOSR Journal of Nursing and Health Sciences.

[15] Mayo Clinic Premature ejaculation.

[16] Falobi O Paraga: Libido boosters or life destroyer?.

[17] Barabara R Why sex is so important to your husband.

[18] Abdullahi H, Tukur J (2013). Sexual stimulants and their effects on women of reproductive age group in Kano, northern Nigeria. Nigerian Journal of Basic Clinical Sciences.

[19] Kaadaaga HF, Ajean J, Ononge S (2014). Prevalence and factors associated with use of herbal medicine among women attending an infertility clinic in Uganda. BMC Complementary and Alternative Medicine.

[20] Odebode AA (2015). Perceived attitudes of health personnel, health care expectations and counselling needs of out-patients of teaching hospitals in Nigeria.

[21] Oniye AO, Odebode AA, Ajape SA (2016). Patterns of aphrodisiac herbs use as expressed by married adults in Kwara State, Nigeria. Nigerian Journal of Guidance and Counselling.

[22] Kamatenesi-Mugisha M, Oryem-Origa H (2010). Traditional herbal remedies used in the management of sexual impotence in Western Uganda. African Health Sciences.

[23] Kubmarawa D, Akiniyi JA, Okorie DA (2013). Ethno medicinal survey of the traditional medicine of Lala people of Nigeria. International Journal of Medicinal Plant and Alternative Medicine.

[24] Orief YI (2012). Use of herbal medicines among pregnant women attending family health centres in Alexandria. Middle East Fertility Society Journal.

[25] Rayner JA, Willis K, Burgess R (2011). Women's use of complementary and alternative medicines: a review of literature. Journal of Altern Complement Med.

[26] Kwara State in Nigeria City population-statistics, maps & charts.

[27] Chowdhury MSI, Ahammed MM, Taher MA, Rahman MA, Ahmed MSAM (2018). Knowledge, attitude and practice towards use of herbal aphrodisiac among Unani & Ayurvedic health care seekers in Dhaka city. Asian J Med Biol Res.

[28] Sand MS, Fisher W, Rosen R, Heiman J, Eardly I (2008). Erectile dysfunction and constructs of masculinity and quality of life in the multinational men's attitude to life events and sexuality. The Journal of Sexual Medicine.

[29] Kalra G, Kamnath R, Subramanyam, Shah H (2015). Psychosocial profile of male patients presenting with sexual dysfunction in a psychiatric outpatient department in Mumbai, India. India Journal of Psychiatry.

